# Analysis of SARS-CoV-2 Transmission in Different Settings, Brunei

**DOI:** 10.3201/eid2611.202263

**Published:** 2020-11

**Authors:** Liling Chaw, Wee Chian Koh, Sirajul Adli Jamaludin, Lin Naing, Mohammad Fathi Alikhan, Justin Wong

**Affiliations:** Universiti Brunei Darussalam, Jalan Tungku Link, Brunei (L. Chaw, L. Naing);; Centre for Strategic and Policy Studies, Jalan Pasar Baru Gadong, Brunei (W.C. Koh);; Ministry of Health, Bandar Seri Begawan, Brunei (S.A. Jamaludin, M.F. Alikhan, J. Wong)

**Keywords:** respiratory infections, severe acute respiratory syndrome coronavirus 2, SARS-CoV-2, SARS, COVID-19, zoonoses, viruses, coronavirus, coronavirus disease, Brunei

## Abstract

We report the transmission dynamics of severe acute respiratory syndrome coronavirus 2 (SARS-CoV-2) across different settings in Brunei. An initial cluster of SARS-CoV-2 cases arose from 19 persons who had attended the Tablighi Jama’at gathering in Malaysia, resulting in 52 locally transmitted cases. The highest nonprimary attack rates (14.8%) were observed from a subsequent religious gathering in Brunei and in households of attendees (10.6%). Household attack rates from symptomatic case-patients were higher (14.4%) than from asymptomatic (4.4%) or presymptomatic (6.1%) case-patients. Workplace and social settings had attack rates of <1%. Our analyses highlight that transmission of SARS-CoV-2 varies depending on environmental, behavioral, and host factors. We identify red flags for potential superspreading events, specifically densely populated gatherings with prolonged exposure in enclosed settings, persons with recent travel history to areas with active SARS-CoV-2 infections, and group behaviors. We propose differentiated testing strategies to account for differing transmission risk.

Cases of coronavirus disease (COVID-19) have escalated since the disease was initially reported on December 31, 2019. A rapid response by the global scientific community has described many aspects of the causative agent, severe acute respiratory syndrome coronavirus 2 (SARS-CoV-2). Estimates suggest a basic reproduction number of 2–3 in the early stages of the outbreak (*1*), which can be valuable in assessing the spread of the virus but obscures individual heterogeneity in the level of infectivity among persons and in different settings (*2*,*3*). Early reports suggest that superspreading events (SSEs) might play a role in the explosive propagation of SARS-CoV-2 (*4*). Targeted approaches that reduce the likelihood of SSEs are contingent on the environmental, behavioral, and host factors that drive transmission and the most effective interventions to control those factors. To address these factors, we report an analysis of a transmission chain in Brunei that resulted from an international SSE.

Brunei is a small, well-connected country in Southeast Asia with a population of 459,500 (*5*). Brunei has multiple land borders and limited state capacity to manage large-scale outbreaks (*6*). Multigeneration households are common and social interactions center on strong family and religious relationships (*7*,*8*). These characteristics make Brunei particularly vulnerable to outbreaks and the rapid progression of clusters to widespread community transmission (*9*).

A COVID-19 case was detected in Brunei on March 9, after a 4-day religious gathering, Tablighi Jama’at, in neighboring Kuala Lumpur, Malaysia. The Tablighi Jama’at gathering in Malaysia has been recognized as an SSE and had >16,000 attendees, including international participants (*10*). Tablighi is an apolitical Islamic movement with adherents from >200 countries. Tablighi adherents usually gather at annual international events lasting several days where they participate in communal prayers, meals, and speeches. In Malaysia, the participants stayed and slept at the mosque, and several of them were deputized to cook and clean. Seventy-five persons from Brunei attended this event. Of the 135 confirmed cases in Brunei reported by the first week of April, 71 (52.6%) cases had an epidemiologic link to this event (Figure 1).

**Figure 1:**
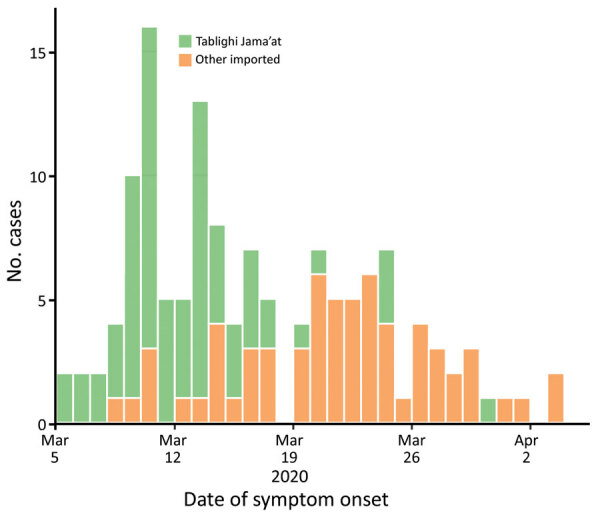
Epidemic curve for the first 135 cases of coronavirus disease (COVID-19) in Brunei Darussalam by cluster groups. Tablighi Jama’at cases were related to a religious gathering in Kuala Lumpur, Malaysia, during February 28–March 1, 2020.

Because SARS-CoV-2 is a novel infection in a naive population, an outbreak investigation of this event can provide insights into its transmission dynamics and the effectiveness of outbreak control measures. Brunei’s thorough contact tracing provides a rare opportunity to study the epidemiologic and transmission characteristics of SARS-CoV-2 in different community settings.

## Methods

### Surveillance and Case Identification

Brunei’s Ministry of Health (MoH) is responsible for communicable disease surveillance and implemented testing criteria for suspected COVID-19 cases on January 23, 2020. Initially, only persons with acute respiratory symptoms and history of travel to a high-risk area were tested for SARS-CoV-2. Over the next several weeks, the program expanded to include contacts of a confirmed case, regardless of symptoms; persons with pneumonia admitted to an inpatient healthcare facility; and persons with acute respiratory illness treated at a health facility for the second time within 14 days. On March 21, MoH started testing and isolating all travelers and returning residents. On March 25, MoH introduced SARS-CoV-2 sampling at selected sentinel health centers to test persons with influenza-like symptoms, and on April 7, MoH implemented mandatory random screening for selected groups of foreign workers.

MoH defined a confirmed COVID-19 case as a person who tested positive for SARS-CoV-2 through real-time reverse transcription PCR (RT-PCR) testing on a nasopharyngeal (NP) swab specimen (*11*). The first positive case in Brunei was detected on March 9 in a person who met the testing criteria by having a fever and cough and having recently traveled to Kuala Lumpur.

### Epidemiologic Investigation

Under the Infectious Disease Act, MoH conducted epidemiologic investigations and collected data for each case and close contact by using the World Health Organization’s first few cases protocol (*12*). The first identified case-patient was interviewed for demographic characteristics, clinical symptoms, travel history, activity mapping, and contact history. Once MoH identified the case-patient’s participation at the Tablighi event in Malaysia, they identified several other persons from Brunei who also had participated at the event. We subsequently obtained the details of all participants from Brunei.

NP swabs were collected from all identified participants and tested with RT-PCR. Persons who tested positive were admitted to the National Isolation Centre (NIC). Persons who tested negative were quarantined for 14 days after their return to Brunei at a designated community quarantine facility, where they were screened for symptoms and body temperature daily. Persons who had symptoms develop at the NIC were retested. Activity mapping of confirmed cases was conducted, and contact tracing was initiated.

We defined a close contact as any person living in the same household as a confirmed case-patient or someone who had been within 1 m of a confirmed case-patient in an enclosed space for >15 minutes (*13*). We identified secondary cases through interviews and checked cellular phone data when information on contacts was uncertain. NP swabs from all close contacts of confirmed case-patients were tested by using RT-PCR. Persons who tested positive were admitted to the NIC and persons who tested negative were placed under home quarantine for 14 days from their last exposure to the confirmed case-patient. Public health workers monitored the compliance and health status of persons under home quarantine daily through video calls or face-to-face assessments. Persons who had symptoms develop during home quarantine were retested.

### Clinical Management

All confirmed case-patients were treated and isolated at the NIC and monitored until recovery. We obtained clinical information on case histories, including any prior treatment by health services, clinical examination, and laboratory and radiological results, from digital inpatient records on the national health information system database. In addition, we collected information on each case-patient’s oral history to ascertain whether they had symptoms ≤14 days before diagnosis. Case-patients were discharged from the NIC after 2 consecutive negative specimens collected ≥24 hours apart.

### Case-Patients

We categorized cases into 2 groups: primary cases were in persons presumably infected at the Tablighi event in Malaysia and nonprimary cases were in persons who had an epidemiologic link to a primary case but did not attend the Tablighi event in Malaysia. For each case-patient, we recorded symptom status and classified them as follows: symptomatic patients reported having symptoms during or before NP swab collection; presymptomatic patients reported having symptoms after NP sampling but during admission to the NIC; and asymptomatic patients reported no symptoms during NP swab collection or admission to the NIC.

### Close Contacts

We classified close contacts into 5 groups or settings: household, relatives, workplace, social, and a local religious gathering. We defined household contacts as persons living in the same household and further classified them by their relationship to a case-patient (spouse, child, or other, which included other familial relationships or housekeepers living in the household). We defined relatives as persons related to a case-patient who lived outside the household, workplace contacts as persons encountered at a workplace or school, and social contacts as those encountered during travel or at social events. We defined contacts from a local religious gathering as persons who attended a local Tablighi event in Brunei on March 5; the event ran throughout the night, and participants stayed all night. Such small local weekly gatherings usually take place among Tablighi adherents in their home countries.

### Data Analysis

We used χ^2^, Fisher exact, or Mann-Whitney tests to compare groups of primary and nonprimary cases, as appropriate. We calculated the incubation period from dates of exposure and symptom onset, when these were clear. We calculated serial interval by subtracting the date of symptom onset of an infectee (secondary case) from the date of symptom onset of the infector (primary case); we only included symptomatic and presymptomatic infector–infectee pairs for which epidemiologic links were clear.

We calculated the attack rate for each setting by dividing the number of positive contacts by the total number of close contacts. To identify risk factors for infection, we applied a log-binomial regression analysis to estimate the risk ratio for gender, age, and setting. We performed further stratification to assess differences in the symptom status of infectors across settings. We estimated the 95% CI by using the normal-approximation method, or the binomial method if the count was <5.

We calculated the mean observed reproductive number (R) and distribution of personal reproductive numbers in each setting by using the number of close contacts infected by each primary case-patient. We estimated the 95% CI by using a Poisson distribution (*14*).

We conducted all analyses by using Excel (Microsoft, https://www.microsoft.com) and R version 3.6.3 (*15*). We considered p<0.05 statistically significant. We obtained ethical approval from the University Research Ethics Committee, Universiti Brunei Darussalam (approval no. UBD/OAVCR/UREC/Apr2020–05).

## Results

### Epidemiologic Characteristics

**Figure 2:**
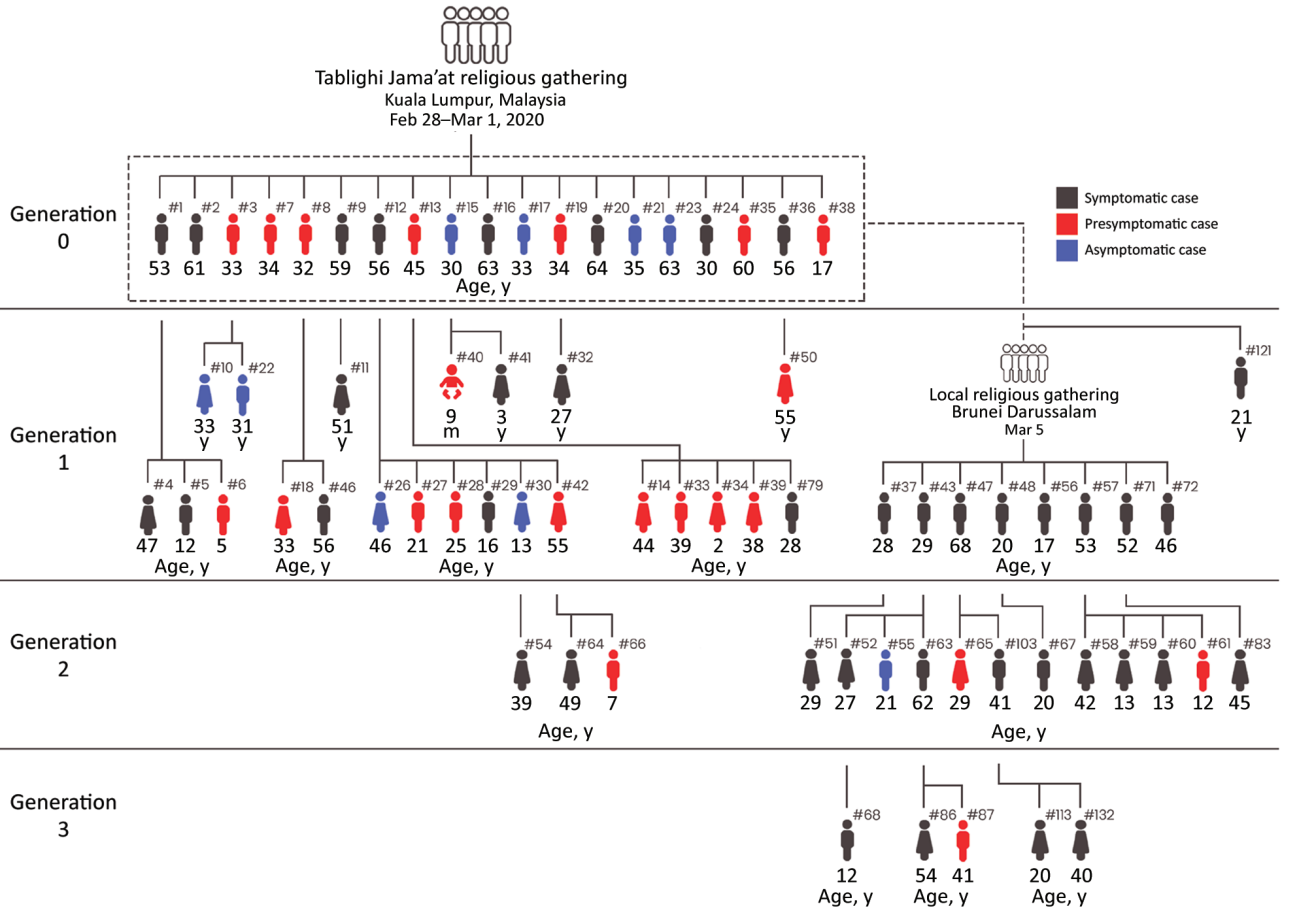
A cluster of coronavirus disease cases in Brunei Darussalam. Epidemiologic links are illustrated by generation and symptomatic status. Generation 0 occurred among attendees of a Tablighi Jama’at gathering in Kuala Lumpur, Malaysia, during February 28–March 1, 2020. Generations 1, 2, and 3 occurred in Brunei. #, case number.

Among 75 persons from Brunei who attended the Tablighi event in Malaysia, 19 tested positive for SARS-CoV-2; 52 local close contacts also tested positive, bringing the total cluster size to 71. We analyzed the epidemiologic links in this cluster by generation in the transmission chain and case-patient symptom status. We noted 32 (45.1%) cases in generation 1, 15 (21.1%) in generation 2, and 5 (7.0%) in generation 3 (Figure 2).

We also analyzed the demographic and clinical characteristics of case-patients in the cluster (Table 1). The median age was 33.0 years (interquartile range [IQR] 21–50 years), 46 (64.8%) case-patients were male and 25 (35.2%) female, and 5 (7.1%) had preexisting chronic conditions. Compared with nonprimary case-patients, primary case-patients were much older, and most were men. Most (55/71; 77.4%) persons with diagnosed COVID-19 were immediately admitted to the NIC within 5 days of symptom onset or NP swab collection (data not shown).

**Table 1:** Demographic and clinical characteristics of cases in a cluster of coronavirus disease among a Tablighi Jamaat community, Brunei

Characteristics	Overall, n = 71	Primary cases, n = 19	Nonprimary cases, n = 52	p value
Median age, y (IQR)	33.0 (21–50)	35.0 (33– 59.5)	29.0 (20–46)	0.009
Range	0.75–68	17–64	0.75–68	
Age group, y				
0–9	4 (5.6)	0 (0.0)	4 (5.6)	0.002
10–19	9 (12.7)	1 (1.4)	8 (11.3)	
20–29	16 (22.5)	1 (1.4)	15 (21.1)	
30–39	14 (19.7)	8 (11.3)	6 (8.5)	
40–49	10 (14.1)	1 (1.4)	9 (12.7)	
50–59	11 (15.5)	3 (4.2)	8 (11.3)	
60–69	7 (9.9)	5 (7.0)	2 (2.8)	
Sex				
F	25 (35.2)	0 (0.0)	25 (48.1)	<0.001
M	46 (64.8)	19 (100)	27 (51.9)	
Underlying conditions				
Obesity	4 (5.6)	2 (10.5)	2 (3.8)	0.289
Heart disease	4 (5.6)	3 (15.8)	1 (1.9)	0.056
Respiratory disease	5 (7.0)	2 (10.5)	3 (5.8)	0.605
Cancer	1 (1.4)	1 (5.3)	0 (0.0)	0.268
Diabetes mellitus	5 (7.0)	3 (15.8)	2 (3.8)	0.115
Symptom status				
Symptomatic	40 (56.3)	8 (42.1)	32 (61.5)	0.265
Median time from symptom onset to diagnosis, d (range; IQR)	4.0 (0–15; 2–6)	3.5 (0–7; 2.75–6)	4.0 (1–15; 2–6.25)	0.746
Presymptomatic	22 (31.0)	7 (36.8)	15 (28.8)	
Median time from symptom to and NP swab collection, d (range; IQR)	0 (−7 to −1; –2.5 to 0)	0 (−1 to 1; 0–1)	0 (−7 to 1; –3 to 0)	0.034
Asymptomatic	9 (12.7)	4 (21.1)	5 (9.5)	
Symptoms ever reported				
Fever	42 (59.2)	9 (47.4)	33 (63.5)	0.343
Cough	42 (59.2)	14 (73.7)	28 (53.8)	0.218
Runny nose	25 (35.2)	7 (36.8)	18 (34.6)	1.000
Sore throat	42 (59.2)	9 (47.4)	33 (63.5)	0.342
Disease severity				
Asymptomatic	9 (12.7)	4 (21.1)	5 (9.6)	0.278
Mild	52 (73.2)	12 (63.2)	40 (76.9)	
Moderate	7 (9.9)	2 (10.5)	5 (9.6)	
Severe or critical	3 (4.2)	1 (5.3)	2 (3.9)	

*Values are no. (%) except as indicated. IQR, interquartile range; NP, nasopharyngeal.

Many case-patients were presymptomatic (22/71; 31.0%) or asymptomatic (9/71; 12.7%) and 40 (56.3%) case-patients reported symptoms during contact tracing investigation. The most reported symptoms were fever, cough, and sore throat. Only 1 (1.4%) case was critical and 2 (2.8%) were severe.

We calculated the incubation period from 8 case-patients who had confirmed epidemiologic links and had attended the March 5 religious gathering in Brunei. By using March 5 as the exposure date, the median incubation period was 4.5 days (range 1–11 days; IQR 2.75–5.5 days). Based on 35 symptomatic infector–infectee pairs, the serial interval was 4.26 days (SD ±4.27 days; range −4 to 17 days). Among the 35 symptomatic infector–infectee pairs, 4 (11.4%) had negative serial interval values. We noted that the serial interval distribution resembled a normal distribution (Appendix Figure 1).

### Transmission Characteristics

Among 1,755 close contacts of the COVID-19 cluster among Tablighi members in Brunei, 52 local transmissions were detected, giving an overall nonprimary attack rate of 2.9% (95% CI 2.2%–3.8%). We excluded case 121 (Figure 2) from the analysis because the case-patient was not detected during contact tracing. The highest attack rates were among spouses (41.9% [95% CI 24.1%–60.7%]), attendees of a local religious gathering (14.8% [95% CI 7.1%–27.7%]), and children (14.1% [95% CI 7.8%–23.8%]). The overall household attack rate was 10.6% (95% CI 7.3%–15.1%).

Multiple log-binomial regression analyses revealed that the type of close contact was the only statistically significant variable (p<0.001; Table 2). Compared with social contacts, spouses of positive case-patients had the highest adjusted risk ratio for infection (45.2 [95% CI 16.8–156.1]), their children had a risk ratio of 14.1 (95% CI 4.8–51.5), and attendees of the local religious gathering had a risk ratio of 15.6 (95% CI 4.8–59.9).

**Table 2:** Risk factors for severe acute respiratory syndrome coronavirus 2 infection among close contacts, Brunei*

Characteristics	Total, n = 1,755	Positive, n = 51	Attack rate, % (95% CI)	Crude risk ratio (95% CI)†	Adjusted risk ratio (95% CI)‡
Sex					
M	913	24	2.6 (1.7–3.9)	Referent	Referent
F	842	27	3.2 (2.2–4.7)	1.22 (0.71–2.11)	1.23 (0.69–2.27)
Age group					
0–9	267	4	1.5 (0.4–3.8)§	Referent	Referent
10–19	163	8	4.9 (2.3–9.8)	3.28 (1.05–12.12)	1.92 (0.63–7.03)
20–29	364	13	3.6 (2.0–6.2)	2.38 (0.85–8.39)	1.91 (0.70–6.61)
30–39	441	6	1.4 (0.6–3.1)	0.91 (0.26–3.53)	0.85 (0.24–3.38)
40–49	255	9	3.5 (1.7–6.8)	2.36 (0.78–8.61)	1.95 (0.63–7.32)
50–59	174	8	4.6 (2.2–9.2)	3.07 (0.98–11.36)	1.84 (0.58–7.05)
≥60	83	3	3.6 (0.8–10.2)§	2.41 (0.48–10.74)	1.00 (0.20–4.51)
Types of close contact					
Social	445	4	0.9 (0.2–2.3)§	Referent	Referent
Relatives	144	5	3.5 (1.3–8.3)	3.86 (1.04, 15.43)	**4.13 (1.10–16.51)**
Local religious gathering	54	8	14.8 (7.1–27.7)	16.48 (5.38–60.13)	**15.60 (4.81–59.87)**
Workplace or school	848	6	0.7 (0.3–1.6)	0.79 (0.23–3.07)	0.79 (0.23–3.10)
Household					
Child	85	12	14.1 (7.8–23.8)	15.71 (5.62–55.16)	**14.09 (4.79–51.54)**
Spouse	31	13	41.9 (24.1–60.7)	46.65 (17.77–158.39)	**45.20 (16.76–156.12)**
Others¶	148	3	2.0 (0.4–5.8)§	2.26 (0.45–10.2)	2.23 (0.44–10.0)

*Bold text indicates statistically significant value. †Calculated by using simple log-binomial regression. ‡Calculated by using multiple log-binomial regression (sex, p = 0.485; age group, p = 0.339; types of close contact, p<0.001). §For counts <5, calculated by using binomial 95% CI. ¶Others include siblings, parents, housekeepers, or relatives, such as grandparents and grandchildren.

Attack rates also differed by symptom status of the infector (Table 3; Appendix Table). In households where the infectors were symptomatic, attack rates were higher (14.4%) than in households in which the infectors were asymptomatic (4.4%) or presymptomatic (6.1%). We could not calculate the attack rate for attendees of the local religious gathering because the 3 primary cases at the event had different symptom statuses and we could not ascertain how transmission occurred. In the household setting, symptomatic case-patients had 2.7 times the risk of transmitting SARS-CoV-2 to their close contacts, compared with asymptomatic and presymptomatic case-patients (crude risk ratio 2.66 [95% CI 1.12–6.34]; Table 3).

**Table 3:** Attack rates in different settings stratified by symptom status of the primary case of severe acute respiratory syndrome coronavirus 2, Brunei*

Setting and symptom status	Total, n = 1,701	Positive, n = 43	Attack rate, % (95% CI)	Crude risk ratio (95% CI)†	p value
Household					
Asymptomatic or presymptomatic	111	6	5.4 (1.2–9.6)	Referent	
Symptomatic	153	22	14.4 (8.8–19.9)	**2.66 (1.12–6.34)**	0.027
Nonhousehold‡					
Asymptomatic or presymptomatic	580	9	1.6 (0.5–2.6)	Referent	
Symptomatic	857	6	0.7 (0.1–1.3)	0.45 (0.16–1.26)	0.129
Overall					
Asymptomatic or presymptomatic	691	15	2.2 (1.1–3.3)	Referent	
Symptomatic	1,010	28	2.8 (1.8–3.8)	1.28 (0.69–2.37)	0.439

*Bold text indicates statistically significant value. † Calculated by using simple log-binomial regression. ‡Includes visits to relatives, workplace, and social settings. The local religious gathering is excluded because 3 primary cases at the event had varying symptom status and we could not ascertain how transmission occurred.

The mean observed R was highest (2.67) among attendees of the local religious gathering. Observed R was 0.67 (95% CI 0.44–0.96) for household members (Table 4). The observed R distribution for the household setting was skewed toward 0 (Appendix Figure 2), and 71.4% (20/28 positive contacts) of household infections were from 16.7% (7/42) of possible links to primary cases.

**Table 4:** Characteristics and mean observed reproductive number for each setting in which infection of severe acute respiratory syndrome coronavirus 2 occurred, Brunei

Setting	No. nonprimary cases	Proportion of links with nonzero infections	Total no. of close contacts	Contacts traced per case	Range of setting size	Mean observed reproductive number (95% CI)
Household	28	0.36	264	9.4	1–13	0.67 (0.44–0.96)
Relatives	5	0.11	144	28.8	1–26	0.26 (0.09–0.61)
Workplace	6	0.20	848	141.3	1–202	0.24 (0.09–0.52)
Social	4	0.16	445	111.3	1–179	0.16 (0.04–0.41)
Local religious gathering	8	1.00	54	6.8	54*	2.67 (NA)*
Overall	51	0.37	1,755	34.4	1–220	0.94 (0.70–1.24)

*Indicates only a single event, so no range for setting size or calculated 95% CI are available. NA, not applicable.

## Discussion

We characterized a cluster of COVID-19 cases in Brunei among attendees of the Tablighi Jama’at in Malaysia, an SSE that led to an epidemic in Brunei. Our analysis revealed several key findings. First, SSEs play a major role in SARS-CoV-2 transmission. Second, transmission variability is high across different settings. Third, transmission varies between symptomatic, asymptomatic, and presymptomatic persons. Our findings highlight the potential for silent chains of transmission.

Within this cluster, 38% of all cases were among participants at an SSE: 19 (26.7%) from the Tablighi event in Malaysia and 8 (11.3%) from a local religious gathering. Of note, 19/75 persons from Brunei who attended the Tablighi event in Malaysia tested positive for SARS-CoV-2. Assuming a representative sample, this suggests an attack rate of 25% and implies that >4,000 of the »16,000 participants at the event in Malaysia might have been infected. Moreover, we found that the highest overall nonprimary attack rate (14.8%) and mean observed R (2.67) were from a local religious gathering, which were higher than the attack rate (10.6%) and mean observed R (0.67) for the household setting. These observations suggest that mass gatherings facilitate SARS-CoV-2 transmission.

During this investigation, we identified several common characteristics at both the local religious gathering and the event in Malaysia (*10*). First, large numbers of attendees gathered in an enclosed area for a prolonged time. Second, some attendees had a history of recent travel; the Tablighi event in Malaysia drew participants from across the world and ≥3 attendees of the local religious gathering had recently returned from Malaysia. Third, the gatherings included communal sleeping areas, sharing of toilet facilities, and shared dining. We propose that these 3 characteristics are hallmarks for an SSE for SARS-CoV-2 transmission. Health authorities can use these characteristics as red flags in their risk assessment and mitigation strategies for preventing and detecting high-risk activities, including mass gatherings, and in other institutional settings, such as care homes, prisons, and dormitories.

To a lesser degree, our observations on the within-household transmission are similar to those observed for the 2 religious gatherings. Among 16 household contacts who subsequently became first generation cases, 10 (62.5%) were from just 3 primary cases. However, even within similar settings, we can expect wide variability in transmission patterns. This observation supports our finding of a moderately high household attack rate but an observed R of <1, suggesting that transmission is driven by a relatively small number of cases (*2*). High attack rates in spouses and children reflect intimate relationships with a high degree of interaction, close proximity, and in the case of the spouse, sleeping in the same room. Concordant with our SSE findings, we suggest that encounters among groups that involve close proximity in enclosed settings for prolonged times (≥1 night) are a main driver of SARS-CoV-2 transmission.

Our overall nonprimary attack rate result of 10.6% in the household setting is comparable to other studies that used contract tracing datasets (*16*; H.-Y. Cheng et al. unpub. data, https://www.medrxiv.org/content/10.1101/2020.03.18.20034561v1; L. Luo et al., unpub. data, https://www.medrxiv.org/content/10.1101/2020.03.24.20042606v1; Q. Bi et al. unpub. data, https://www.medrxiv.org/content/10.1101/2020.03.03.20028423v3). A study near Wuhan, China (*17*), reported a higher attack rate of 16.3%, but they detected 56.2% of cases >5 days after persons began having symptoms. By contrast, 77.4% of cases in our study were detected and patients were isolated ≤5 days of symptom onset, suggesting that early case isolation can reduce the attack rate. The Brunei MoH’s strategy of aggressive testing of contacts might have contributed to reduced attack rates among household members.

We noted a low nonprimary attack rate (<1%) and mean observed R (<0.3) for workplace and social settings. Moderate physical distancing was implemented in Brunei following the identification of this cluster, but community quarantine and lockdown were not implemented. Public services and businesses remained open and no internal movement restrictions were imposed in the country.

Combined with our observations on the role of SSEs in driving SARS-CoV-2 transmission, we suggest that areas with limited community transmission can avoid full lockdown measures that adopt a blunt approach of restricting all movement. Instead, such areas can use a more targeted approach that combines case isolation, contact tracing, and moderate levels of physical distancing and takes into account the red flags for mass gatherings we identified. However, this approach is resource intensive and only feasible in communities with sufficient public health capacity. The high proportion of asymptomatic persons suggests that even with best efforts at contact tracing, the potential for widespread community transmission is clear. Once SARS-CoV-2 is established in a location, its suppression requires implementation of broader physical distancing measures (*18*,*19*). Nonetheless, effective contact tracing and case isolation approaches have been shown to control COVID-19 during the early stage of outbreaks (*20*). In addition, modeling studies using data from South Korea showed that less extreme physical distancing measures can help suppress an outbreak (*21*).

We identified several environmental settings and behavioral factors that potentially account for higher attack rates observed in mass gatherings and households. To assess the effect of host factors in driving transmission, we compared the nonprimary attack rate in symptomatic, asymptomatic, and presymptomatic persons, considering the high proportion of asymptomatic (12.7%) and presymptomatic (31.0%) case-patients. Case reports of presumptive asymptomatic and presymptomatic SARS-CoV-2 transmission have been published (*22*,*23*), but few observational studies quantify such transmissions. A study from Ningbo, China, analyzed the overall attack rates between symptomatic and asymptomatic COVID-19 case-patients and did not find major differences between the 2 groups (*24*). Another study reinterpreted the same data and theorized that SARS-CoV-2 could be more transmissible from symptomatic than asymptomatic persons under certain conditions (*25*). In fact, our overall crude risk ratio for symptomatic case-patients showed no statistically significant difference compared with asymptomatic or presymptomatic case-patients (Table 3; Appendix Table). However, we suggest this finding masks the true picture in transmissibility when different settings are taken into account.

We did not find statistically significant differences in the attack rate for nonhousehold settings, which usually practice some form of nonpharmaceutical interventions (NPI), such as taking medical leave for persons with moderate or severe symptoms. In addition, some physical distancing likely would be practiced by contacts of persons with visible symptoms. However, our findings suggest that transmission occurs more frequently at the household level where such physical distancing and control measures are less practical. We observed that the household attack rate for symptomatic persons (14.4%) is higher than that of asymptomatic (4.1%) or presymptomatic (6.1%) persons, suggesting that presence of symptoms is a host factor in driving transmission.

The higher household attack rate observed among symptomatic case-patients suggests that testing for contacts of symptomatic persons should be prioritized, especially in low resource areas. Nonetheless, the attack rates we observed for asymptomatic (4.4%) and presymptomatic (6.1%) case-patients were not negligible and our findings have several implications for high resource areas with greater testing capacity. First, it strengthens the argument for testing household contacts in the absence of symptoms. Second, some flexibility should be permitted in the surveillance system because the high proportion of asymptomatic case-patients poses challenges for rapid detection and isolation. Thus, we recommend that moderate levels of physical distancing should be implemented even in countries with highly developed testing and tracing capacities. Third, proactive testing of travelers, attendees of red flag events, and persons housed in institutional settings might be necessary to contain COVID-19 spread.

This study has several limitations. First, because we conducted a retrospective study based on a contact tracing dataset, determination of the index case and direction of transmission could be uncertain, particularly because a substantial proportion of case-patients were asymptomatic. Moreover, we did not account for outside sources of infection, so setting-specific attack rates could have been overestimated even though no community transmission has been detected in Brunei. Viral sequencing can confirm homology between the strains infecting index and secondary cases across the various settings but was not conducted for all cases. Second, we have not accounted for other potential environmental factors, such as the relative household size, time spent at home with others, air ventilation, and transmission from fomites. Third, we do not have information on NPIs practiced by close contacts; presumably, persons would take precautions during an outbreak. Fourth, case-patients reported their symptom status during NP swab collection, which we assumed to be reflective of their condition when their close contacts were exposed; however, this might not be true for all cases. Finally, the generalizability of our results is limited because there was no community transmission, the small number of cases, and the lack of cases in communal settings, such as residential care facilities and dormitories.

The main strength of our study is the availability of a complete contact tracing dataset at the national level. Because all case-contacts were tested, we believe our study more accurately describes SARS-CoV-2 transmission than studies in which only symptomatic case-contacts were tested.

In conclusion, our analysis highlights the variability of SARS-CoV-2 transmission across different settings and the particular role of SSEs. We identify red flags for potential SSEs and describe environmental, behavioral, and host factors that drive transmission. Overall, we provide evidence that a combination of case isolation, contact tracing, and moderate physical distancing measures can be an effective approach for SARS-CoV-2 containment.

Appendix Additional information on transmission of severe acute respiratory syndrome coronavirus 2, Brunei.
