# Supplementary material for: Analysis of SARS-CoV-2 Transmission in Different Settings, Brunei
Source: Emerg Infect Dis. 2020 Nov;26(11):2598–606. doi: 10.3201/eid2611.202263 (PMC7588541; doi:10.3201/eid2611.202263)
Supplement: Appendix — Additional information on transmission of severe acute respiratory syndrome coronavirus 2, Brunei. [file 20-2263-Techapp-s1.pdf]

Article DOI: https://doi.org/10.3201/eid2611.202263

# Analysis of SARS-CoV-2 Transmission in Different Settings, Brunei

## Appendix

**Appendix Table.** Attack rates and risk ratio in different settings, stratified by symptom status of the infector

| Settings | Total (n = 1,701) | Positive (n = 43) | Attack rate, % (95% CI)* | Crude risk ratio† (95% CI) | p value |
|---|---|---|---|---|---|
| Household | | | | | |
| Asymptomatic | 45 | 2 | 4.4 (0–10.5) | 1.00 | |
| Presymptomatic | 66 | 4 | 6.1 (0.3–11.8) | 1.36 (0.26–7.13) | 0.713 |
| Symptomatic | 153 | 22 | 14.4 (8.8–19.9) | 2.66 (0.79–13.24) | 0.102 |
| Nonhousehold‡ | | | | | |
| Asymptomatic | 61 | 1 | 1.6 (0–4.8) | 1.00 | |
| Presymptomatic | 519 | 8 | 1.5 (0.5–2.6) | 0.94 (0.12–7.39) | 0.953 |
| Symptomatic | 857 | 6 | 0.7 (0.1–1.3) | 0.43 (0.05–3.49) | 0.427 |
| Overall | | | | | |
| Asymptomatic | 106 | 3 | 2.8 (0–6.0) | 1.00 | |
| Presymptomatic | 585 | 12 | 2.1 (0.9–3.2) | 0.72 (0.21–2.52) | 0.613 |
| Symptomatic | 1,010 | 28 | 2.8 (1.8–3.8) | 0.98 (0.30–3.17) | 0.972 |

*Attack rate (incidence) with normal approximation 95% CI.
†Calculated by simple log-binomial regression.
‡Nonhousehold exposures occur among relatives, and in the workplace social settings. The local religious gathering is excluded here because 3 primary cases at the event had varying symptom status; we could not ascertain how transmission occurred.

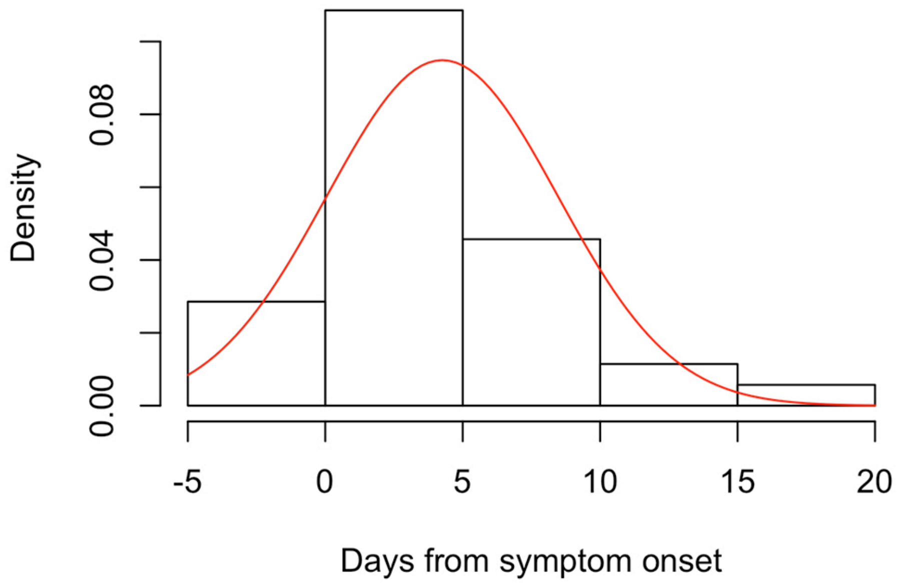


**Appendix Figure 1**. Distribution of the serial interval of cases of severe acute respiratory syndrome coronavirus 2 (SARS-CoV-2), Brunei. Red line represents normal distribution curve.

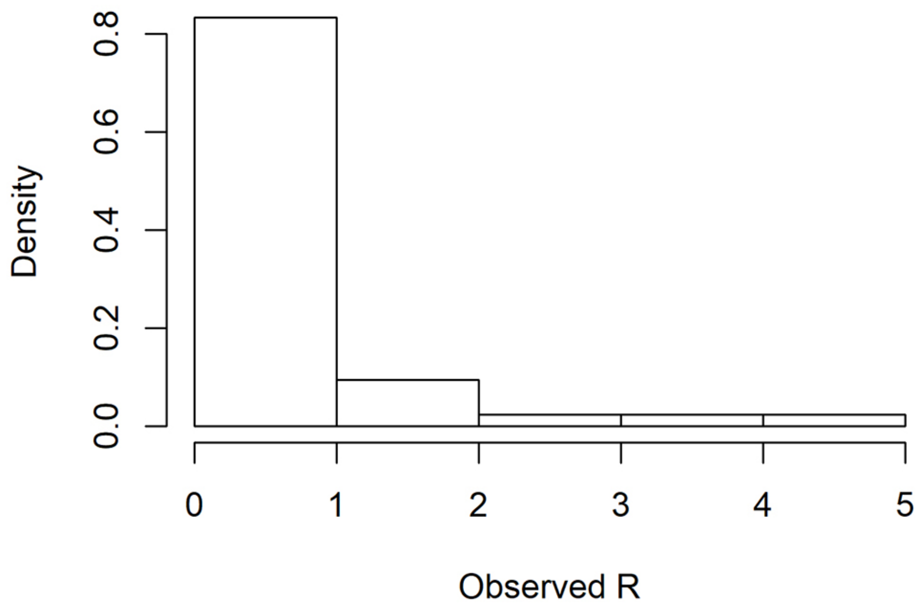


**Appendix Figure 2**. Distribution of the observed reproductive number (R) among household members of primary cases.
